# Quantification of Carbonyl Groups in Lignin: ^31^P NMR after Reduction

**DOI:** 10.1021/acssuschemeng.5c06762

**Published:** 2025-09-04

**Authors:** Nicolò Pajer, Claudia Crestini

**Affiliations:** † Department of Molecular Sciences and Nanosystems, Ca’ Foscari University of Venice, Via Torino 155, Venezia-Mestre, Venice 30155, Italy; ‡ Centre for Colloid and Surface Science, Via della Lastruccia 3, Sesto Fiorentino, Florence 50019, Italy

**Keywords:** lignin, lignin structural characterization, biorefinery, carbonyl groups, quantitative analyses, ^31^P NMR spectroscopy

## Abstract

The available analytical techniques for the determination of carbonyl groups in lignins suffer from several drawbacks including tedious protocols and the need for highly powerful NMR spectrometers for acquiring processable-quality spectra in short times. In the present effort, these limitations are overcome by introducing a protocol based on the quantitative reduction of carbonyl groups, followed by the quantification of the resulting increase in hydroxyl groups by ^31^P NMR spectroscopy. The methodology, yielding results that align with the oximation technique and quantitative ^13^C NMR data, has been optimized on technical (hardwood and softwood kraft lignins and wheat straw organosolv lignin) and analytical-grade lignins (acidolysis lignins and enzymatically mild acidolysis lignins). This approach, when coupled with HSQC data, also allows for the identification of the nature of different carbonyl groups in the analyzed lignins. All in one, quantitative ^31^P NMR after sodium tetrahydroborate reduction constitutes a reliable and straightforward analytical protocol for the identification and quantification of carbonyl groups in lignin.

## Graphical abstract

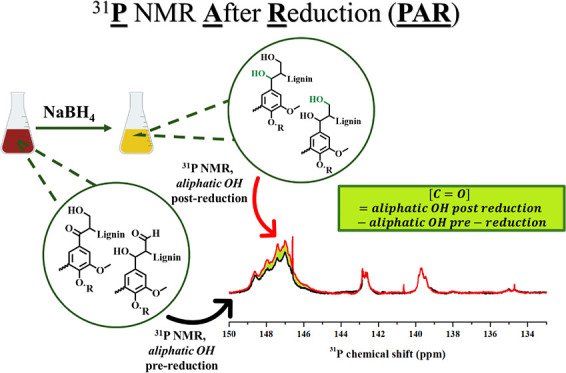

## Introduction

Developing effective strategies for the valorization of lignin necessitates a thorough and accurate quantification of its various functional groups as these groups play a crucial role in determining the chemical reactivity and potential applications of lignin-derived products. From this perspective, the possible role of carbonyl groups has been, so far, largely underestimated.^1–4^

Lignin is characterized by the presence of non-negligeable amounts of carbonyl groups, which may appear under different forms, both on the propanoic side chains, and on the aromatic rings.^5^ These structures belong to three major classes: aldehyde, ketone, and quinone groups^6^ (Figure 1). Cinnamaldehydes and benzaldehyde groups appear in lignin both as a result of its natural biogenesis and upon extractive or specific functionalization processes.^7–12^ Ketonic groups can be of both Hibbert-ketone-nature (on the α- and β-positions, on hydroxylated-side chains, structures a and b in Figure 1), typically derived from acidolysis procedures, or on the α-position of the propanoic side-chain, originated mostly via oxidative pathways (e.g., enzymatic transformations).^13–16^ Additionally, small amount of quinones can be found,^6,17,18^ typically as the result of pulping conditions associated with demethylation processes.

**Figure 1:**
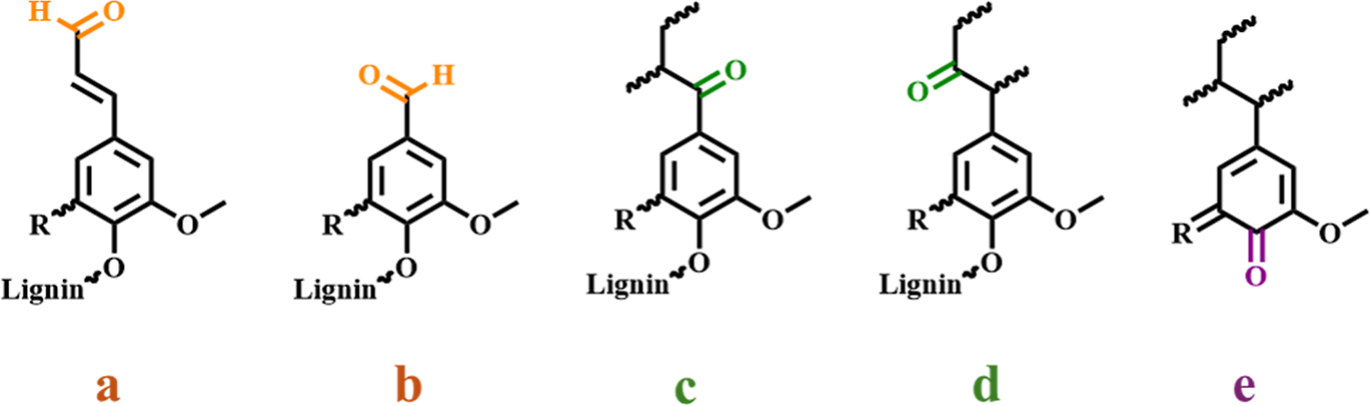
Typical carbonyl groups in lignins; (a,b) aldehydic (respectively, cinnamaldehyde and vanillin-like), (c,d) ketonic (including Hibbert ketones), and (e) quinonoid.

The estimation of the structural features of lignins, such as the content of typical bonding patterns like aryl-glycerol-β-aryl ethers or phenyl-coumarans, as well as functional groups like hydroxy or methoxy groups, is commonly performed using NMR. This can involve both monodimensional (traditional ^1^H and ^13^C, or advanced ^31^P NMR after phosphitylation)^19–25^ or two-dimensional (semiquantitative HSQC or, way more better for rigorously quantitative analyses, HSQC_0_)^26,27^ techniques. However, considering the precise estimation of carbonyl groups in lignin, the use of traditional NMR spectroscopy remains challenging.

^1^H NMR spectra allow for the identification of lignin aldehydes groups, which are characterized by well-resolved peaks in the chemical shift range between 9.56 and 9.94 ppm,^19,28–31^ excluding the possible estimation of ketonic carbonyls.^28–30^ On the other hand, quantitative ^13^C NMR is a promising technique for quantifying these functional groups as it allows their detection and differentiation in the chemical shift range between 180 and 210 ppm, a well-resolved region of the lignin spectra.^32–35^ However, the overall procedure for quantitative ^13^C NMR spectra is plagued by several limitations: (a) long acquisition times, especially due to the long relaxation times of quaternary carbons; (b) the weakness of the carbonyl group signals; and (c) the relatively high field strength required (at least 500 MHz, based on proton resonance frequency) to reduce the number of transients needed to obtain a processable-quality spectrum. Additionally, the validity of ^13^C to quantify carbonyls was questioned since the accuracy of this method is limited.^36^ In the case of two-dimensional NMR techniques, their utility is even more restricted. By excluding the unique homocorrelated INADEQUATE experiments, which observe the coupling of vicinal ^13^C atoms—practically unfeasible for the analysis of nonisotopically labeled lignins^37,38^ —no heterocorrelated experiments are capable to directly observe carbonyl groups.^35^ Consequently, any indirect observation and quantification of these groups can be regarded as dubious and unreliable, especially given the significant differences in relaxation times between various moieties.

To address these limitations of traditional NMR analyses, labeling techniques for carbonyl groups based on fluorination protocols which allow carbonyls quantification by exploiting the NMR resonances of inserted heteroatoms have been developed.^39,40^ Specifically, carbonylated moieties are functionalized with a fluorine-containing agent (e.g., via hydrazones); the resulting derivatives are then analyzed using ^19^F NMR in the presence of a fluorinated internal standard. Although these methodologies hold considerable potential, their applicability has been limited since their development (respectively, 1999 and 2001), primarily due to the complex derivatization procedures involved.

As an alternative to NMR-based quantification of carbonyl groups in lignin, wet-chemistry methodologies have been developed, relying on quantitative chemical transformations to obtain analytical data.^41^ Reduction-based methods monitor the chemical reduction of lignins in organic solvents via UV–vis spectroscopy, specifically measuring the variation in absorbance,^42^ or using the so-called gasimetric-method,^43,44^ by measuring the volume of hydrogen released from the unreacted reducing agent. Oximation-based techniques, on the other hand, take advantage of the selective addition of hydroxylamine to carbonyl groups, resulting in the formation of oximes.^44,45^ The oximation procedure involves the administration of a known amount of hydroxylamine hydrochloride to the lignin sample; the hydrochloric acid released from the reaction is then titrated and accounts for the carbonyl groups content.

Both the reduction-based and the oximation-based methodologies require not only a properly trained operator but also high precision in weighing operations, as well as during titration and data processing (e.g., the creation of Δε curves). This results in high experimental errors and a low reliability of the obtained data.

All in one, to date, there is a lack of a simple, globally standardized methodology for the quantitative determination of carbonyl groups in lignins. All of this highlights the urgent need for a fast and reliable procedure to obtain data on carbonyl groups amount and nature in lignin.

The present effort reports a simple and straightforward NMR-based procedure for the determination of aldehyde and ketone carbonyl groups in lignin based on ^31^P NMR after reduction (PAR). This analytical procedure consists of the determination of the increase of aliphatic hydroxy groups in lignin after its quantitative reduction with sodium tetrahydroborate as determined by ^31^P NMR. An overall structural perspective on the optimized reduction of lignins is discussed in detail (based on gel permeation chromatography and HSQC data), demonstrating that it does not affect any other features of lignin than carbonyl groups. Differently from the use of quantitative ^13^C NMR, this approach does not require the use of a strong magnetic field NMR spectrometer (200 MHz NMR spectrometers allow the acquisition of well-resolved spectra), the acquisition time for the spectra is extremely fast (less than 30 min), and the reduction of carbonyls is straightforward, easy, and quantitative. The results obtained with PAR are in full agreement with those obtained using the oximation procedure and quantitative ^13^C NMR in the presence of an internal standard as well as data reported in literature.

## Experimental Part

Acetovanillone, acetosyringone, vanillin, syringaldehyde, sodium hydroxide, *N*-hydroxy-5-norbornene-2,3-dicarboxylic acid imide (NHND), and lithium chloride were purchased from Sigma-Aldrich and used with no additional purification. Ethanol, methanol, dioxane, and anhydrous pyridine were obtained in puriss. p.a. quality from Sigma-Aldrich. Deuterochloroform, perdeuterated dimethyl sulfoxide, and perdeuterated pyridine were purchased from Sigma-Aldrich, with deuteriation percentage above 99.8%. 1-Chloro-4,4′,5,5′-tetramethyl-1,3,2-dioxaphospholane (TMDP) was synthesized in the laboratory, purified via double vacuum-distillation, and characterized via ^31^P NMR demonstrating a spectroscopic purity above 99%.

### Dioxane Lignin Isolation

30 g of acetone/water (9:1) exhaustively extracted Wiley milled Loblolly Southern Pine or White Oak wood were refluxed under the nitrogen atmosphere, after presoaking, in 600 mL of 0.2 N hydrochloric acid in dioxane/water for 2 h. The resulting mixture was filtered; the precipitate was washed three times with fresh dioxane, neutralized with finely ground sodium bicarbonate, and concentrated to a final volume of ∼50 mL. The solution was added dropwise in 1 L of 0.2% sodium sulfate solution, and the precipitated lignin was allowed to coagulate overnight at 4 °C. Precipitated lignin was isolated via centrifugation (15 min, 5000 rpm). The latter was thoroughly washed with distilled water up to negative reaction of litmus paper as well as negative chloride and sulfate tests. The resulting lignin was air-dried and finally dried in a vacuum-oven set at 40 °C.

### Enzymatic Mild Acidolysis Lignin Isolation

The present lignin was isolated from acetone/water (9:1) exhaustively extracted and ball-milled White Oak wood according to the protocol developed by Argyropoulos using Sigma-Aldrich cellulases.^46,47^

### Reduction of Lignin Model Compounds

Reduction tests were conducted on the ketonic group (acetovanillone and acetosyringone) and the aldehyde group containing models (vanillin and syringaldehyde). 1 mmol model was dissolved in 10 mL of a solvent mixture composed of 0.1 N sodium hydroxide, ethanol, and dioxane (volume ratio 1:4:1; in the case of acetosyringone, methanol was used in lieu of ethanol for solubility issues) in a 50 mL Erlenmeyer flask. Subsequently, 200 mg (5.3 mmol) of sodium tetrahydroborate was added in small portions to the solution. The resulting solution was gently stirred at room temperature for 24 h. Afterward, additional 40 mg of sodium tetrahydroborate (1.1 mmol) was added to the mixture and the latter was stirred again for additional 24 h. The resulting solution was acidified with 1:5 hydrochloric acid to a final pH of 3 and extracted three times with 20 mL aliquots of a suitable extracting solvent (ethyl acetate, diethyl ether, methylene chloride, or chloroform). Organic phases were combined, dried over sodium sulfate, filtered, and concentrated to dryness under reduced pressure at 40 °C. The resulting product was finally dried overnight in a vacuum oven set at 40 °C and analyzed via the standard ^31^P NMR protocol further described.

### Optimization of the Reduction Conditions

1.00 g of lignin was dissolved in 60 mL of a freshly prepared solvent mixture composed of 0.1 N sodium hydroxide, ethanol, and dioxane (volume ratio 1:4:1) in a 125 mL Erlenmeyer flask. 500 mg of sodium tetrahydroborate (13.2 mmol) was administered to the mixture in small portions; the resulting solution was then stirred for 24 h at room temperature. Additional portions of 100 mg of sodium tetrahydroborate (2.6 mmol) were added to the mixture in the same manner in the following days up to a maximum total addition of 800 mg of reducing agent. The resulting mixture was transferred in a 220 mL centrifuge bottle, and reduced lignin was precipitated via acidification using 0.1 N hydrochloric acid (final pH 3, no more hydrogen/borane evolve from the solution). The reaction mixture was diluted to 200 mL with distilled water, and lignin was isolated via centrifugation (15 min, 5000 rpm). The resulting lignin was washed via dispersion in water/centrifugation up to a neutral reaction of litmus paper. Afterward, the resulting lignin was freeze-dried and vacuum-dried at 40 °C.

Blanks were prepared under otherwise identical conditions by keeping lignin solutions under stirring for the entire duration of the reduction; finally, lignin was isolated after acidification and washings.

### Analytical Reduction

300 mg of lignin was dissolved in 20 mL of a freshly prepared solvent mixture composed of 0.1 N sodium hydroxide, ethanol, and dioxane (1:4:1 v/v/v) in a 40 mL Erlenmeyer flask. The solution was split in two aliquots in 30 mL flasks, one for the blank and the other for the reduction. The solution aliquot for the blank was kept stirring at room temperature for the entire duration of the reduction treatments. The solution aliquot for the reduction was administered with 40 mg of sodium tetrahydroborate (1.1 mmol); the mixture was stirred at room temperature for 24 h. Afterward, 10 mg of sodium tetrahydroborate was added to the reducing solution, and the latter was stirred for additional 24 h. The operation is repeated for an additional time. Blank and reduced mixtures were transferred with little amounts of ethanol in 50 mL vials and acidified with 0.1 N hydrochloric acid (final pH around 3, no more hydrogen/borane evolved from the solution); the same volume of hydrochloric acid used for the reduced mixture was used for the blank. Both mixtures were made up to 45 mL with distilled water, and the precipitated lignins were isolated via centrifugation (15 min, 5000 rpm). The lignins were washed via dispersion in water/centrifugation up to neutral reaction of the supernatant upon test with litmus paper. Resulting samples were freeze-dried and vacuum-dried at 40 °C.

### ^31^P NMR Spectra Acquisition

^31^P NMR spectra were acquired according to the original procedure described by Argyropoulos with slight modifications.^20,48^ In particular, 20 mg of vacuum-dried lignin preparation was dissolved in 400 μL of a pyridine/CDCl_3_ mixture (1.6:1 v/v) with additional 70 μL of a chromium­(III) acetyl acetonate solution (5 mg/mL, in 1.6/1 pyridine/CDCl_3_). Afterward, 70 μL of internal standard solution (NHND 0.126 M, in 1.6/1 pyridine/CDCl_3_) was added. The mixture was stirred at room temperature resulting in a clear solution; to the latter, 70 μL of TMDP was added. The solution was rapidly transferred to a NMR tube, and the ^31^P NMR spectrum was recorded no later than 2 h after the addition of TMDP. Spectra were acquired at room temperature using a Bruker 300 MHz NMR spectrometer; standard inverse-gated proton-decoupled pulse sequence with 90° flip angle was used. 128 transients were acquired per each sample each with a pulse delay of 12 s. Spectral processing as well as integrations were performed using MestReNova according to standard practices.^20,35^

### Carbonyl Estimation via Oximation

The determination of carbonyl groups was performed with a modified version of the original oximation protocol.^49,50^ Potentiometric titration was used in lieu of high-frequency titration. In a 20 mL crimpable-vial, 80 mg of vacuum-dried lignin was dissolved in 2 mL of analytical grade dimethyl sulfoxide; to the resulting solution, 5 mL of an oximating mixture—constituted of hydroxylamine hydrochloride 0.2 M in a 0.08 M triethanolamine solution in water/ethanol—was added. The vial was flushed with nitrogen, sealed with a Teflon cap, and the mixture was incubated at 80 °C for 2 h. After the vial was cooled to room temperature, the content of the vial was quantitatively transferred to an Erlenmeyer flask by the use of a little amount of distilled water. The solution was potentiometrically titrated with a standardized 0.100 N hydrochloric acid solution to a final pH of 3.3. A blank with no lignin was run at the same time. The amount of the carbonyl group was expressed in mmol per gram using the following eq 1:

1 $$carbonyl\;groups\;\left(mmol/g\right)=\frac{\left(V_{0}-V_{l}\right)\cdot 0.100}{m_{lignin}}$$

where *V*_0_ and *V*_l_ represent, respectively, the volume of 0.100 N hydrochloric acid utilized for the titration of the blank and the lignin sample, expressed in milliliters, while *m*_lignin_ is the mass of the analyzed lignin, in grams.

### ^13^C Spectra Acquisition

Quantitative ^13^C NMR spectra of lignins were recorded according to the protocol devised by Xia in the presence of 1,3,5-trioxane as an internal standard.^25^ Specifically, 200 mg of lignin was dissolved in 400 μL of d^6^-dimethyl sulfoxide. Subsequently, 50 μL of 0.100 M chromium­(III) acetylacetonate in d^6^-dimethyl sulfoxide was added as a relaxation agent, followed by 200 μL of a 0.800 M 1,3,5-trioxane solution in the very same solvent. The solution was transferred to an NMR tube and analyzed in a Bruker 300 MHz NMR spectrometer. Prior to the acquisition of the quantitative ^13^C spectra, relaxation times of the samples were measured via a standard Bruker inversion recovery pulse sequence. Quantitative ^13^C spectra were acquired for all the samples at 50 °C with the inverse-gated proton-decoupled pulse sequence with 90° flip angle; 18 k transients were acquired with an acquisition time of 0.5 s and a pulse delay of 12 s. Spectral processing as well as integrations were performed according to the protocol previously described^35^ using MestReNova.

### HSQC Analyses

The HSQC sample preparation as well as the spectral processing were performed as previously described.^35^ The pulse-sequence used and quantitative measurements were made according to the quantitative methodology described by Zhang and Gellerstedt.^51^ Spectra were recorded using a Bruker Advance 400 MHz NMR spectrometer.

### GPC Analyses

The GPC analyses were performed according to protocols previously published.^16,52,53^ Specifically, a 1 mg/mL lignin solution in dimethyl sulfoxide was eluted at 70 °C by a 0.1% lithium chloride solution in HPLC-grade dimethyl sulfoxide in a Shimadzu HPLC system equipped with an Agilent PLgel 5 μm MiniMIX column. A photodiode-array detector was employed as the detector; by the use of a calibration curve made of standard polystyrene-sulfonates (Sigma-Aldrich), the molecular weights as well as the dispersion indexes of the samples were determined.

## Results and Discussion

### Principle of the Methodology

The quantitation of carbonyl groups in a lignin sample is performed by estimating the increase in the content of aliphatic hydroxyl groups after their quantitative and selective reduction with a mild reducing agent, sodium tetrahydroborate.

Under these experimental conditions, ketones are converted into secondary and benzylic hydroxy groups, while aldehydes are transformed into primary hydroxyl groups (Scheme 1).

**Scheme 1:**
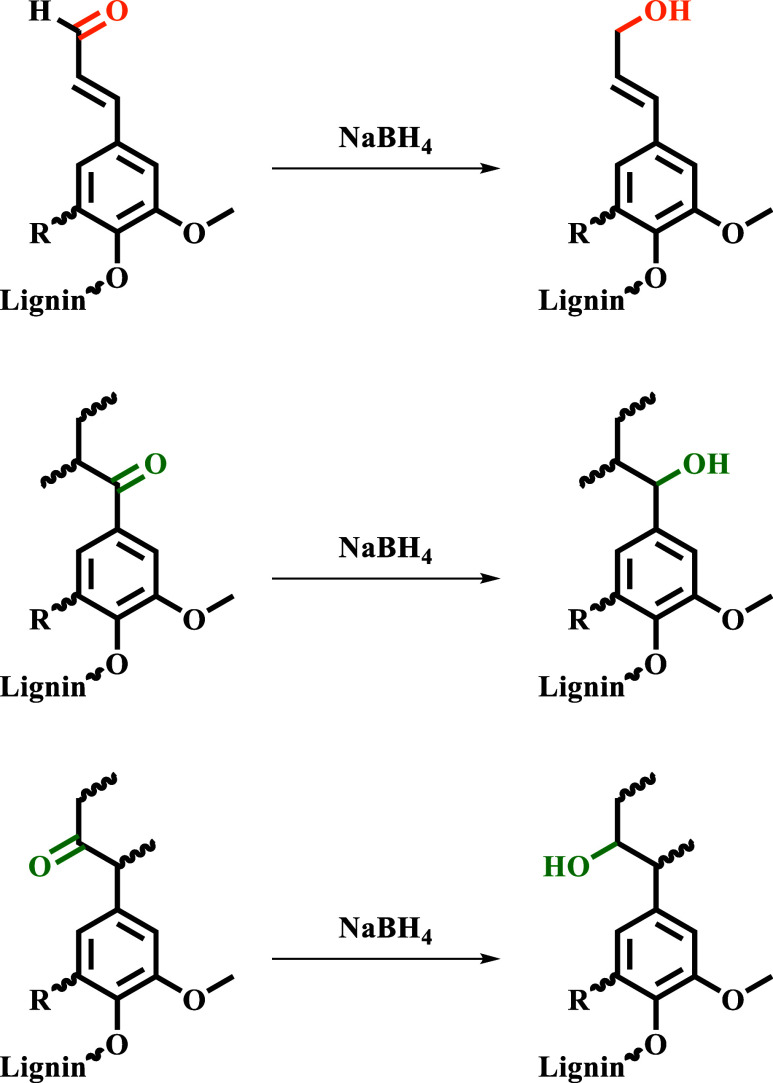
Reduction of Aldehyde and Ketone Carbonyls in Lignin

^31^P NMR spectroscopy is, to date, the most precise, reliable, and fast analytical technique for the determination of hydroxyl groups in lignin. Not only after P-labeling the spectra can be acquired using a spectrometer operating with a magnetic field not necessarily high (200 or 300 MHz instruments provide good data) but also the increasingly popular 70–80 MHz benchtop NMR instruments yield excellent results.^54^ Therefore, this technique has been applied to the quantitative determination of the increase of aliphatic hydroxyl groups after reduction. This approach, when compared to the quantification of hydroxy groups by quantitative ^13^C NMR, is evidently more convenient owing to the faster acquisition times, the need of unsophisticated NMR spectrometers, and to a simpler and in situ derivatization process.^22,23^

Specifically, in ^31^P NMR after reduction (PAR), both the starting lignin and the reduced sample are phosphitylated in pyridine/deuterochloroform by using TMDP (Scheme 2) in the presence of a known amount of internal standard (NHND). Then, the ^31^P NMR spectra of the resulting solutions are recorded. The overall acquisition time is around 30 min.

**Scheme 2:**
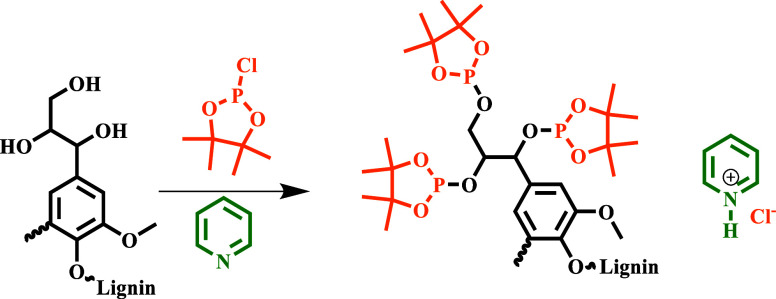
. TMDP-Based Phosphitylation of Lignin

The content of carbonyl groups is finally given by eq 2:

2 $$carbonyl\;groups\;\left(mmol/g\right)={aliphatic\;OH}_{after\;reduction}\;\left(mmol/g\right)-{aliphatic\;OH}_{before\;reduction}\;\left(mmol/g\right)$$

where aliphatic OH_after reduction_ (mmol/g) and aliphatic OH_before reduction_ (mmol/g) are, respectively, the content of aliphatic hydroxyl groups in the sodium tetrahydroborate-reduced sample and in the unmodified sample. Figure 2 depicts the ^31^P NMR spectrum of the same lignin before and after the sodium tetrahydroborate reduction. The increase in the signal between 149 and 146 ppm, corresponding to aliphatic hydroxyl groups, reflects the reduction of carbonyl groups present in the starting material.

**Figure 2:**
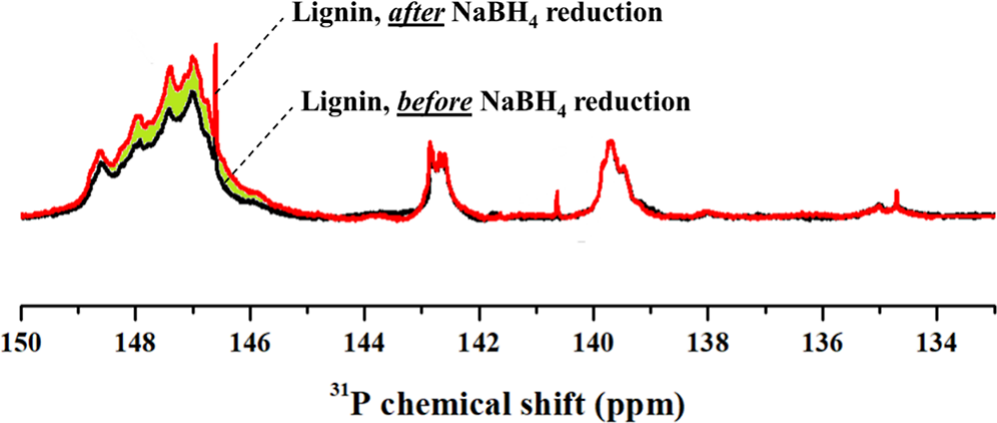
Overlapped ^31^P NMR spectra of the same lignin before and after quantitative sodium tetrahydroborate reduction.

### Reduction of Lignin Model Compounds by Sodium Tetrahydroborate

The development of the methodology started with the screening of the reactivity of lignin model compounds bearing typical lignin aldehydic or ketonic groups. Specifically, acetovanillone (**1**) and acetosyringone (**2**) were elected as reference for ketonic groups, while vanillin (**5**) and syringaldehyde (**6**) were chosen as model substrates for the aldehydic groups (Scheme 3).

**Scheme 3:**
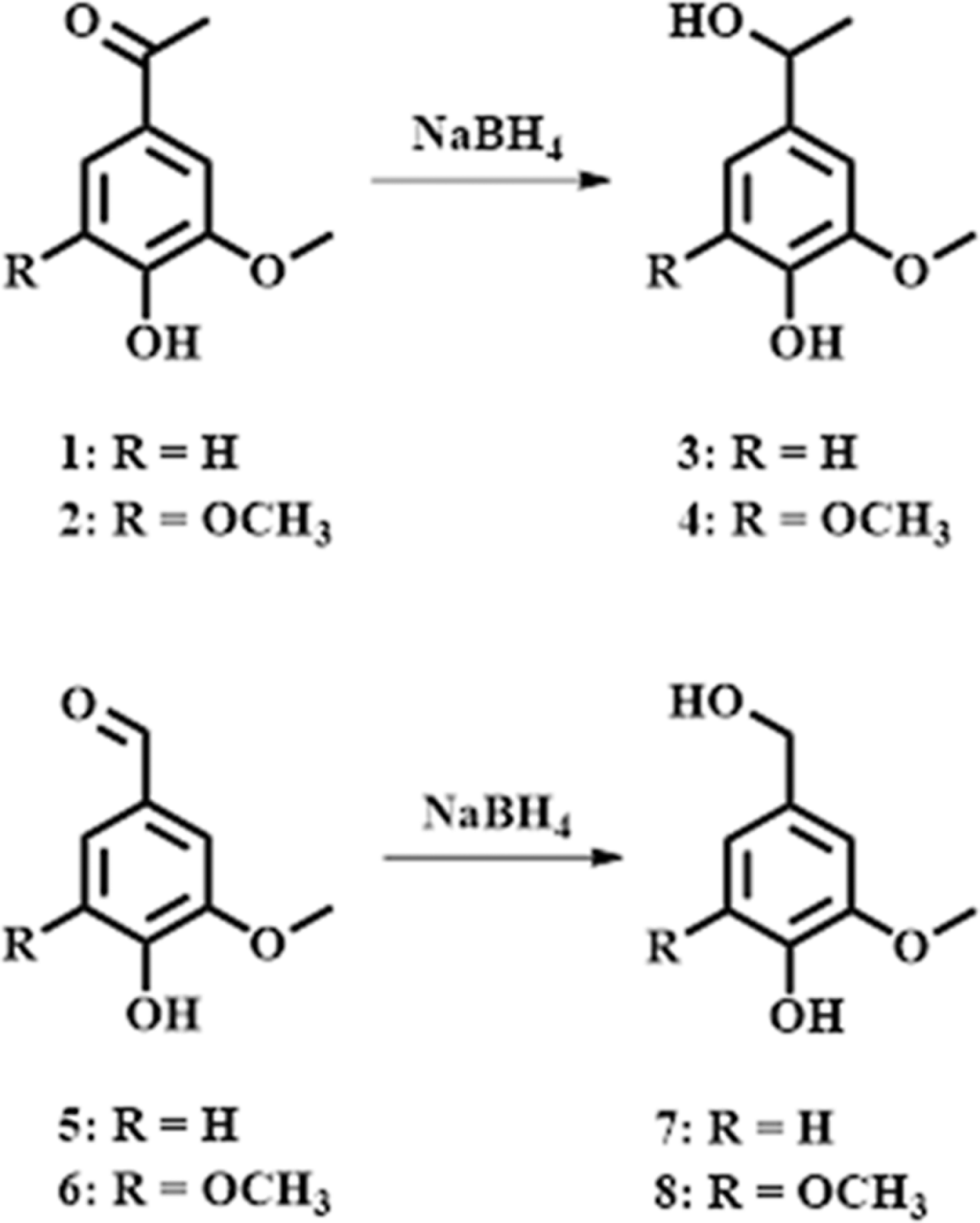
Reduction of Model Compounds[Fn s3fn1]

a
**1**: acetovanillone; **2**: acetosyringone; **5**: vanillin; **6**: syringaldehyde.

The chosen model compounds are characterized by the presence of one phenolic hydroxyl group per carbonyl group; consequently, in a fully reduced sample, the ratio between phenolic and aliphatic, deriving from the reduction process, hydroxyl groups is 1. In view of that, the yield of the reduction was calculated from the ^31^P NMR spectra according to the following eq 3:

3 $${reduction}_{yield}\;\left(\%\right)=\frac{{area}_{aliphatic\;OH}}{{area}_{phenolic\;OH}}\cdot 100$$

where area_aliphatic OH_ and area_phenolic OH_ are, respectively, the areas of the peaks pertaining to the aliphatic and the phenolic hydroxyl groups of the model after phosphitylation with TMDP. Reduction yields are summarized in Table 1.

**Table 1:** Reduction Yields of Model Compounds^a^

	model	product	reduction yield (%)
ketone models	1	3	96 ± 3
	2	4	98 ± 4
aldehyde models	5	7	99 ± 3
	6	8	96 ± 4

a The NMR spectra were run in triplicate.

By considering the high yield achieved as well as the intrinsic potential integration error in the spectral processing, the sodium tetrahydroborate capability to quantitatively reduce lignin-related carbonyl compounds was demonstrated.

### Reduction of Lignin by Sodium Tetrahydroborate

The sodium tetrahydroborate reduction of lignin has been previously reported in studies with different aims with divergent protocols. In relatively recent times, Sevillano reported that the dissolution, or the dispersion, of lignin in a mixture composed of ethanol and dioxane and the administration of a large excess of sodium tetrahydroborate (mass ratio lignin/sodium tetrahydroborate (lig/NaBH_4_) 1:1) followed by a 48 h incubation resulted in the full reduction of the sample.^40^ However, the use of a largely alkaline environment may result in hydrolytic processes liberating hydroxy groups from ester and ether bonds which, especially in the present case, where a precise determination is targeted, may compromise the final analytical result. Therefore, the optimal reduction process should imply only the necessary amount of reducing agent to afford quantitative conversion, added with particular care to the reaction mixture.

Another relevant issue is lignin solubility to ensure a quantitative and homogeneous reduction, the lignin sample should be completely solubilized in the reaction mixture. During the present investigation, it was found particularly difficult to dissolve some technical lignins (e.g., steam explosion lignin) in the reductive reaction medium reported by Sevillano, showing the need to identify a better solvent system to perform the reaction.

In this perspective, the reduction process was optimized targeting the identification of (a) a suitable solvent system to perform the reaction and (b) the best methodology to quantitatively reduce the sample avoiding large excess of sodium tetrahydroborate.

#### Choice of the Solvent System

Short-chain alcohols (C1–C3) did not ensure good solubility, and 1-butanol was excluded due to issues in its removal from the reaction products. Dioxane failed as well; aqueous dioxane (9:1) or alcohols solution in water were excluded due to the hydrolyzing activity of water toward sodium tetrahydroborate. A ternary mixture composed of a 0.1 N sodium hydroxide solution, ethanol, and dioxane 1:4:1 (v/v/v), referred to as “alkaline solvent”, was identified as an optimal candidate for a wide range of lignin preparations (kraft, organosolv, steam explosion, dioxane acidolysis, and enzymatically mild acidolysis). The alkalinity deriving from sodium hydroxide ensured the deprotonation of the samples, favoring their dissolution in the ethanolic medium; dioxane, with its optimal Hildebrand parameter,^55,56^ co-operated enhancing the overall solubility. The alkalinity of the alkaline solvent has also an additional advantage; in fact, it preserves sodium tetrahydroborate from being hydrolyzed into hydrogen and trioxoborates (III) by water.^57^

The reduction of lignin was optimized on the basis of an earlier protocol involving the treatment of lignin with an initial excess of sodium tetrahydroborate (lig/NaBH_4_ 1:0.50) for 24 h and by further additions of another smaller aliquot, lig/NaBH_4_ 1:0.20, followed by an incubation of additional 24 h.^58^ In view of that, it was decided to identify the optimum amount of sodium tetrahydroborate to be used for preparing a fully reduced lignin, avoiding unnecessary excesses. Three technical lignins were considered as benchmark-references for the present optimization step: a softwood kraft lignin (SKL), a hardwood kraft lignin (HKL), and a wheat straw organosolv lignin (OL). Specifically, lignin dissolved in alkaline solvent was prereduced with sodium tetrahydroborate in lig/NaBH_4_ 1:0.50 (w/w); then, smaller aliquots of sodium tetrahydroborate in lig/NaBH_4_ 1:0.10 ratio were administered each after 24 h of incubation. Samples prepared at different reducing agent loads were isolated (lig/NaBH_4_ 1:0.50, 1:0.60, 1:0.70, and 1:0.80). The content of aliphatic hydroxyl groups for each sample was estimated via ^31^P NMR, and the respective content of reduced carbonyls was calculated according to eq 2, reported in the Principle of the Methodology section.

Details on the spectral processing operations for ensuring good reproducibility of the results as well as the spectra of the starting and fully reduced lignins (Figure S1) are provided in the Supporting Information. With regard to the integration of the peaks, the very same range of chemical shifts must be used for both pristine and reduced samples. In this regard, it is recommended to compare the spectra, identify a suitable range of chemical shifts including all of the signals of aliphatic hydroxyl groups for all samples, and then apply it to all of them. In all the performed analyses for the present research, a chemical shifts ranged between 145.5 and 149.5 ppm was demonstrated to be suitable.

The comparison of the carbonyl contents obtained at different lig/NaBH_4_ loads, determined via PAR, is listed in Table 2.

**Table 2:** Content of Carbonyl Groups Estimated via Oximation, ^13^C NMR, and PAR on SKL, HKL, and OL

		content of carbonyl groups (mmol/g)		
		SKL	HKL	OL
oximation		0.58 ± 0.03	0.47 ± 0.03	0.31 ± 0.03
quantitative ^13^C NMR		0.54 ± 0.08	0.43 ± 0.09	0.29 ± 0.08
reduction followed by ^31^P NMR (PAR)	1-step reduction: lig/NaBH_4_ 1:0.50	0.35 ± 0.02	0.38 ± 0.02	0.18 ± 0.02
	2-steps reduction: (1) lig/NaBH_4_ 1:0.50 (2) lig/NaBH_4_ 1:0.10	0.54 ± 0.03	0.43 ± 0.03	0.26 ± 0.02
	3-steps reduction: (1) lig/NaBH_4_ 1:0.50 (2) lig/NaBH_4_ 1:0.10 (3) lig/NaBH_4_ 1:0.10	0.53 ± 0.03	0.42 ± 0.03	0.29 ± 0.02
	4-steps reduction: (1) lig/NaBH_4_ 1:0.50 (2) lig/NaBH_4_ 1:0.10 (3) lig/NaBH_4_ 1:0.10 (4) lig/NaBH_4_ 1:0.10	0.55 ± 0.03	0.44 ± 0.03	0.29 ± 0.03

As expected, SKL, HKL, and OL were not completely reduced after the initial reduction treatment using lig/NaBH_4_ 1:0.50, as demonstrated by the fact that the administration of an additional amount of sodium tetrahydroborate resulted in a notable increase in the measured carbonyl groups. For both SKL and HKL, a plateau in the content of carbonyl group, estimated via PAR, was reached after the subsequent administration of an additional lig/NaBH_4_ 1:0.10 aliquot (total amount of sodium tetrahydroborate added to 1.00 g of lignin, 600 mg (15.8 mmol)). Negligible fluctuations of around ±0.01 mmol carbonyl groups per gram of lignin were observed administering two more lig/NaBH_4_ 1:0.10 aliquots. Consequently, it was concluded that the optimal sodium tetrahydroborate reduction of SKL and HKL was obtained by a prereducing step achieving the conversion of the majority of carbonyl groups, followed by an additional step, permitting to reduce also more recalcitrant structures. This was particularly evident in the case of OL, which required two additional aliquots of sodium tetrahydroborate for achieving a quantitative reduction of its carbonyls. In fact, as previously reported, carbonylated structures like α-guaiacoxy-propiovanillone (1-(4′-hydroxy-3′-methoxyphenyl)-2-(2-methoxyphenoxy)-propan-1-one) are particularly resistant to the action of sodium tetrahydroborate, requiring up-to 70 h to achieve the full reduction.^42^ In an additional experiment, in which sodium tetrahydroborate was administered to a SKL solution in alkaline solvent in a lig/NaBH_4_ 1:0.60, instead of a two-steps reduction (lig/NaBH_4_ 1:0.50 + lig/NaBH_4_ 1:0.10), with a 24 h incubation time, a lower amount of carbonyl groups was measured via the PAR method (0.41 ± 0.03 mmol/g), confirming the existence of these recalcitrant patterns which cannot be fully reduced in just a 24 h-treatment, even if in the presence of an excess of sodium tetrahydroborate.

### On the Need of Blanks for PAR

The effect of alkaline solvent on SKL, HKL, and OL was considered in view of the possible structural variations that may occur on the samples during alkaline incubation. The latter may translate in intrinsic variation in the content of hydroxylated moieties, independent from the sodium tetrahydroborate reduction, that overall impact the estimation of carbonyls via PAR. In view of that, the content of aliphatic hydroxyl groups of pristine lignins (SKL, HKL, and OL) was compared to the one of a blank, run under the very same conditions, prepared without the reducing agent. Results demonstrated that a slight decrease in the content of aliphatic hydroxyl groups between 2 and 4% was measured. These results align with previous findings for both kraft and organosolv lignins.^16^ Since the experimental error in hydroxyl groups determination via ^31^P NMR is of approximately 8%, the quantified variation may be considered as negligible. In this respect, it seemed logical that there is no need to prepare a blank to consider the effect of alkaline solvent.

In a wider perspective, the very same conclusions cannot be applied to all samples. In fact, lignins having high amounts of carbohydrate contaminations, like lignin-carbohydrate complexes, contain unnegligible amounts of labile ether and ester bonds that are easily cleaved under mild alkaline conditions, even at room temperature. The present fact is corroborated by early findings demonstrating that carbohydrate-containing lignin samples from different sources hydrolyze under mildly alkaline conditions undergo a decay process liberating monosaccharidic fragments.^59,60^ As released fragments contain hydroxyl groups, their removal from the analyte affects the overall estimation of aliphatic hydroxyl groups in the sample for PAR, resulting in unreliable quantification of carbonyl groups. Consequently, under these circumstances, it is recommended that one should consider a blank. It can be prepared simply by dissolving the sample in the alkaline solvent at the same concentration of the sample utilized for the reduction, keeping it under incubation at room temperature for the whole duration of the reduction treatment, and finally processing it as the reduced sample. In the present case, the formula utilized for estimating the content of carbonyl groups will be (eq 4):

4 $$carbonyl\;groups\;\left(mmol/g\right)={aliphatic\;OH}_{after\;reduction}\;\left(mmol/g\right)-{aliphatic\;OH}_{blank}\;\left(mmol/g\right)$$

where aliphatic OH_blank_ (mmol/g) represents the content of aliphatic hydroxyl groups in the blank.

### Methodology Validation

In order to validate the present methodology, the carbonyl content estimated via PAR for SKL, HKL, and OL was compared to the one measured by the oximation methodology in ethanolic triethanolamine and dimethyl sulfoxide, as previously reported^49,50^ (results are reported in Table 2).

PAR applied to fully reduced samples is an accurate technique for the quantification of aldehydic and ketonic carbonyl groups on lignins, as demonstrated by the comparison of the results to those achieved via oximation. In order to ensure the total reduction of the sample used as references as reduced-lignins to be used for the carbonyl estimation via PAR, the oximation was applied to them, revealing that only minimal amounts of carbonyl, in line with the experimental error, were measured.

A minor discrepancy, comprising between 0.02 and 0.04 mmol/g, was observed while comparing the carbonyl content estimated via PAR and by oximation. In this respect, the impact of quinonoid groups (Figure 3) was considered.

**Figure 3:**
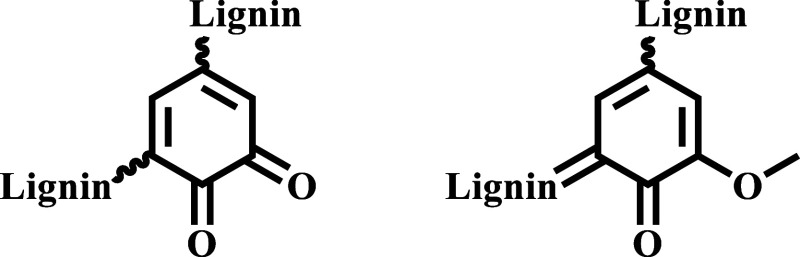
Typical quinonoid groups in lignins.

These functional groups are not quantified by the PAR as their reduction products (catechols, but in general phenols) are not included in the considered quantification range of the hydroxyl group formed (aliphatic hydroxyl groups). On the opposite, during carbonyl determination via oximation, quinonoid systems are converted into the respective enamines and the hydrochloric acid liberated from the reaction is titrated along with the one formed from aldehydic and ketonic groups, so they contribute to the total carbonyl content. A confirmation of the possible role of quinones on the gap between PAR and oximation results was found in the NMR-based quantification of quinones by ^31^P NMR, which demonstrated that common technical lignins are characterized by the quinoid content comprised between 0.01 and 0.03 mmol/g.^61^ The reported values are aligned with the observed discrepancy.

Quantitative ^13^C NMR analyses of the samples were performed to support the validity of PAR as a reliable methodology for estimating carbonyls in lignin as well as to corroborate the hypothesis of its selectivity for quantification of aldehydic and ketonic carbonyls. Spectra were recorded in the presence of 1,3,5-trioxane as an internal standard as devised by Xia,^25^ after determining, via the inversion recovery experiment, the optimal pulse delay to ensure the complete relaxation of the nuclei to the original Boltzmann distribution prior to repeat the excitation cycle. The sum of the areas of the peaks appearing in the range comprised between 210 and 190 ppm, pertaining to aldehydic and ketonic carbonyls, normalized on the basis of the area of the internal standard (93 ppm) was used to calculate the total carbonyl groups content of the sample.^35^ By avoiding considering the carbonyl-peaks characterized by chemical shifts below 190 ppm,^22^ quinones were excluded in this quantification. The data obtained via PAR align with those obtained via quantitative ^13^C NMR data (Table 2), corroborating the validity of PAR as a reliable method for the selective quantification of nonquinonoid carbonyls. In addition, it was possible to confirm the quantitative reduction of carbonyls by performing quantitative ^13^C NMR analyses of the reduced lignins. Specifically, the disappearance of the peaks appearing in the range between 210 to 190 ppm was considered as indicative of the quantitative process. A pictorially emphasized comparison (Figure S2) of the quantitative ^13^C spectra of SKL before and after the quantitative reduction is shown in the Supporting Information.

### Semimicro Scale Reduction and Its Application to Analytical Grade Lignins

A semimicro scale protocol was optimized to perform analyses on a 300 mg scale—for samples requiring a blank (e.g., lignin-carbohydrate complexes of carbohydrate highly contaminated lignins, e.g., steam explosion or enzymic)—or 150 mg—for sample not requiring a blank. Reduction steps are performed in the very same manner of the macro-scale process, with scaled-down amounts of sodium tetrahydroborate. In order to avoid weighting and adding small amounts of sodium tetrahydroborate (10 mg) for the final reduction steps, the use of a sodium tetrahydroborate solution in 0.1 N sodium hydroxide can be efficient as well. In this respect, it is fundamental that the solution is freshly prepared as the reducing agent easily degrades.

In the semimicroscale process, by directly transferring the reduction mixture (or the blank) (∼10 mL) in centrifuge Falcons prior to the acidification, it was observed that the yields are increased. After freeze-drying and vacuum-drying, reduced lignins are obtained in the range between 100 and 120 mg. Since at least 20.0 mg of lignin are required to run a ^31^P NMR quantitative analyses of hydroxyl groups, this amount is optimal. In addition to that, the high yields in reduced lignins ensure that even less experienced operators can obtain suitable amounts of material for the needed analyses at least in triplicate (60 mg).

The comparison of the content of carbonyl groups via PAR using both the semimicro- and the macro-scale procedures is summarized in Table 3. The optimized semimicro scale reduction yielded the same results within the experimental errors, confirming its validity.

**Table 3:** Content of Carbonyl Groups (mmol/g) Estimated via Macro-Scale and Semimicro Scale PAR on SKL, HKL, and OL

	SKL	HKL	OL
macro-scale	0.53 ± 0.03	0.42 ± 0.03	0.26 ± 0.02
semimicro scale	0.52 ± 0.03	0.43 ± 0.03	0.28 ± 0.02

The semimicroscale PAR protocol was applied to three analytical grade lignins. Specifically, dioxane lignins from Loblolly Southern Pine and White Oak as well as White Oak enzymatically mild acidolysis lignin were utilized. As in the case of both dioxane lignins, unnegligible amounts of carbohydrates were found in the samples (Klason acid insoluble lignin contents, respectively, of 89 and 78%); the final content of carbonyl was calculated by considering a blank sample.

In the present cases, reduced lignins appeared to be less soluble in the pyridine/deuterochloroform mixture employed for the spectroscopic analysis than blanks. The situation changed after the addition of the TMDP for both White Oak (dioxane lignin and enzymatically mild acidolysis lignin); in fact, the phosphitylation permitted to obtain clear solutions suitable for the ^31^P NMR analyses. In the case of dioxane lignin from Loblolly Southern Pine, a cloudy solution resulted even after the addition of TMDP; the addition of little amounts of *N*,*N*′-dimethyl-formamide prior and after the TMDP-treatment did not result in an enhancement of the solubility of the preparation. In the present circumstances, a homogeneous solution was obtained using the solvent system composed of *N*-ethyl-*N*-methyl-imidazolium chloride: *N*,*N*′-dimethylformamide—pyridine.^62^ Results for the content in carbonyl groups of these lignins are given in Table 4.

**Table 4:** Content of Carbonyl Groups (mmol/g) Estimated via Semimicro Scale PAR on Loblolly Southern Pine Dioxane Lignin, White Oak Dioxane Lignin, and White Oak Enzymatically Mild Acidolysis Lignin

	carbonyl content (mmol/g)
Loblolly Southern Pine dioxane lignin	0.94 ± 0.01
White Oak dioxane lignin	1.07 ± 0.03
White Oak enzymatically mild acidolysis lignin	0.77 ± 0.01

These results demonstrated a relevantly higher content in carbonyl groups for dioxane lignins and enzymatically mild acidolysis lignin than those of the previously analyzed technical lignins. This difference is due to the different nature of the samples. In the case of enzymatically mild acidolysis lignin, which is generally recognized for being close in terms of structure to the native lignin existing in plants,^46,47^ the presence of a higher amount of carbonyl groups if compared to technical lignins aligns with early findings on lignin structures. Specifically, Adler and Gierer^63^ as well as Bjorkman and Peterson,^64^ respectively, reported that the contents of carbonyl groups of native lignins from Spruce and Pinus Abies are of 0.5 and 0.2 carbonyl groups per methoxy group. Similar to Adler and Gierer, Gierer and Soderberg measured 0.48 carbonyl groups per methoxy group in spruce lignin.^42^

The higher content of carbonyl groups in dioxane lignins was attributed to the effect of the acidolysis processes occurring during the extraction; in fact, the acidic decay is known to lead to mild degradation processes causing lignin depolymerization liberating phenolic groups and contemporary generating carbonyl species on the α- and β-position of the side chains (Hibbert ketones).^5,65,66^

### Insights on the Sodium Tetrahydroborate Reduction of Technical Lignins

The HSQC analyses of the samples before and after the quantitative reduction permitted not only to conclude that sodium tetrahydroborate affects only carbonyl groups—suggesting that other minor processes simultaneously occurring do not cause under- or over-estimation of their content via PAR—but also to tentatively elucidate the nature of certain carbonyl groups present in the analyzed lignins.

The HSQC spectra (Figures S3–S5 in Supporting Information) were recorded on fully reduced samples and were compared to those of pristine samples. Results of the semiquantitative analyses of the functional groups based on 100 aromatic units, using the G_2_^13^C–^1^H signal as internal standard, are summarized in Table 5.

**Table 5:** Content of Lignin Subunits in SKL, HKL, and OL before (Pristine) and after (Reduced) Sodium Tetrahydroborate Reduction (% of G_2_^13^C–^1^H unit)^a^

		SKL		HKL		OL	
		pristine	reduced	pristine	reduced	pristine	reduced
oxygenated signals	A	5	5	6	4	30	38
	B	2	2	0	1	5	6
	C	5	5	7	8	0	0
	VA		4				
	D				2		
	I	0	4	0	0	0	0
	J	1	0	1	1	0	0
aromatic signals	G_2_	100	100	39	40	56	42
	G_5_	117	101	47	49	106	107
	G_6_	117	101	33	31	30	23
	S_2,6_			61	60	80	79
	S/G			0.78	0.76	0.71	0.70
	H_2,6_	6	6			7	7
	T_6_					7	7
	T′_2,6_					6	7
	*p*CE_2,6_					6	6
	FA_7_					10	10

a A: aryl-glycerol-β-aryl ether, B: phenylcoumaran, C: resinols, I: cinnamyl alcohol, J: α-etherified lignin-carbohydrate complexes, VA: α-carbon in vanillic alcohols, D: α,β-diaryl-glycerol; G: guaiacyl ring, S: siringyl ring, H: *p*-hydroxy-phenyl ring; T: tricin, *p*CE: *p*-coumaric acid esters; FA: ferulic acid esters.

In the case of SKL, a relevant aspect was represented by the appearance in the spectrum of reduced-SKL of a novel peak at 4.70/63.3, which was completely absent in pristine SKL. This peak was assigned to the C_α_ of vanillyl alcohol, suggesting the existence of vanillin residues in SKL.

With regard to the aryl-glycerol-β-aryl bonding pattern (A), no relevant variations were observed in the content of the various carbons constituting its aliphatic side chain. This allowed us to exclude the presence of carbonyl groups on A_α_, which would have become evident after reduction increasing the content of A_α_. Additionally, no terminal formyl groups on this bonding pattern were noticed as their presence would have been confirmed by an increase in the content of the A_γ_ signal.

The analyses of the content of phenylcoumaran (B) and resinol (C) demonstrated the overall validity of the sodium tetrahydroborate reduction conditions utilized as a not-impacting process on the nature of aliphatic bonds in lignins. Sodium tetrahydroborate did not result in the ring-opening process of the heterocyclic rings in phenyl-coumaran and resinol systems, which would have been resulted in an increase in the content of the B_α_, B_β_, and all C signals. As no B_γ_ signals were revealed in pristine SKL as well as in its reduced form, the presence of terminal formyl groups on phenyl-coumaran was excluded. Their reduction would have resulted in the formation of primary hydroxyl groups on B_γ_ leading to the appearance of the B_γ_ signal in the spectrum.

A noticeable increase in the intensity of the signal pertaining to cinnamyl alcohol (I) was noticed from SKL, where a negligible amount was detected in the starting lignin, indicating the existence of cinnamaldehyde residues in the starting lignin, which upon reduction were converted into primary alcohols. No alterations on the content of lignin-carbohydrate complexes content (J_α_) were noticed, preserving the overall features of the sample.

With regard to the aromatic signals of SKL, a slight reduction in the content of G_5_ was observed upon sodium tetrahydroborate treatment, possibly due to coupling processes which may lead to the generation of new diaryl-ethers. This hypothesis is supported by the observed decrease of the guaiacyl signal of the ^31^P NMR spectra of both the blank and SKL after reduction. Clearly, the alkaline solvent pH conditions used are responsible for such a modification. As this process does not affect carbonyl group content, it was concluded that it did not affect the overall analytical determination. Gel permeation chromatography data supported the slight polymerization of the sample (Figure S6, Supporting Information), demonstrating a mild effect of the alkaline solvent by comparing pristine SKL with the blank as well as a reduced sample.

For HKL, bonding patterns of A-type did not relevantly change in their content upon reduction, supporting the previous finding discussed for SKL demonstrating the overall lack in the content of α-carboxy-arylglycerol-β-aryl structures. The appearance of a weak B_α_ signal in the HSQC spectrum of reduced HKL, previously absent in pristine HKL, did not represent a relevant modification due to its poor intensity (1 B_α_ per 100 aromatic units); the latter was attributed to the mere intensification of a very weak signal threshold excluded in the case of pristine HKL. Similar conclusions were drawn for the overall intensification of the signals pertaining to the C-type bonding patterns.

The appearance of a distinct peak in the reduced HKL HSQC spectrum at 5.51/75.2 ppm suggests the existence of the α-carbon of the α,β-diaryl-propan-1,2-diol (β,1′) bonding pattern (Table 5 signal D). A second signal, at 3.49/58.8 ppm, tentatively assigned to the D_β_ via spectral simulation, supports the present hypothesis. The D_γ_ signal is expected to be characterized by a chemical shift included in the typical range of B_γ_; this made challenging its unvocal assignment. The lack of the D_α_ signal in the HSQC spectrum of pristine HKL indicates that the β,1′ structure exists in the original lignin under an “HSQC-masked Ca-oxidised form”(α-carboxy-α,β-diaryl-propan-3-ol). The β-carbon in the present structure, according to spectral simulation, is characterized by a chemical shift close to the one of the D_β_ justifying the appearance of the present signal in the spectrum of pristine HKL; its intensity decreased after the reduction, thus, corroborating the present hypothesis (Scheme 4).

**Scheme 4:**
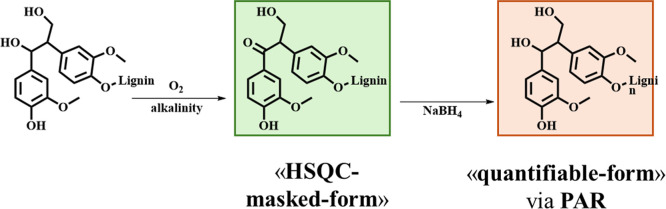
Sodium Tetrahydroborate Reduction of α-Carbon-Oxidised D-Pattern, Corresponding to Its “HSQC-Masked α-Carbon Oxidised Form” Yields the D Pattern Quantified by PAR and Directly Detected by HSQC

The origin of β,1′ pattern in lignin is traditionally attributed to lignin intrinsic biogenesis^67^ and recondensation processes occurring during the kraft cooking.^68–70^ In all cases, the alkaline-α-carbon-oxidation of β,1′deriving from kraft pulping yields the α-carbon-oxidized D-pattern.

In accordance with the results obtained for SKL samples, a slight variation in the content of guaiacyl and syringyl C–H signals was observed in the aromatic area coupled with a decrease in the measured S/G ratio. These variations aligned with the data obtained from quantitative analyses of phenolic hydroxyl groups via ^31^P NMR. GPC analyses of pristine HKL and its reduced counterpart and the comparison of the ^31^P NMR and GPC data (Figure S7, Supporting Information) with the blank supported of a mild alkaline-induced polymerization only deriving from the reaction environment.

Finally, OL was demonstrated to contain carbonyl groups mainly on the aryl-glycerol-β-aryl ether structures. An increase in the A_α_ signal after reduction clearly revealed the existence of benzylic ketones (e.g., propiophenones) in the original sample. The same trend was found for the A_γ_ signals, suggesting the presence of terminal phenyl-propanoic aldehydes. With regard to the variation in the A_β_ intensity, this increase was more limited if compared to those pertaining to other A-structures, suggesting the existence of only limited amounts of ketonic-carbonyls. The nature of the latter may most-likely be attributed to Hibbert ketones, which are formed by means of the extraction due to acidity of organosolv process.^5,65,66^

A relevant aspect taken into consideration while comparing the spectra of OL before and after sodium tetrahydroborate reduction was represented by the content of ferulates (FA) and *p*-coumarates (*p*CE). These structures, that are abundant in grass lignins,^71,72^ are known for being quantitatively hydrolyzed under alkaline conditions—at room temperature in 1 M sodium hydroxide.^26,73^ As a direct consequence of this process, the liberation of hydroxyl groups from the alkoxy residue, especially those of aliphatic type, may alter the results in estimating the carbonyl group content by the PAR method.

To rule out this possibility—especially owing to the alkalinity of the alkaline solvent—special attention was paid to quantify any potential decrease in the content of CE and FA. HSQC spectra unequivocally revealed that no quantifiable variation in their content had occurred (Table 5). This finding is further supported by the quantification of carboxylic hydroxyl groups before and after reduction by ^31^P NMR. Specifically, in the case of hydrolysis—as described by the equation shown in Scheme 5—a notable increase in the content of these groups would be expected. However, numerical data showed that their concentrations remained constant in the original lignin (0.30 mmol/g) and in the reduced sample (0.30 mmol/g), thereby confirming the absence of hydrolytic processes. This outcome was attributed to the relatively mild alkalinity of the environment ([NaOH] ∼ 0.30 mol/L), which was insufficient to hydrolyze these bonding patterns (Scheme 5).

**Scheme 5:**
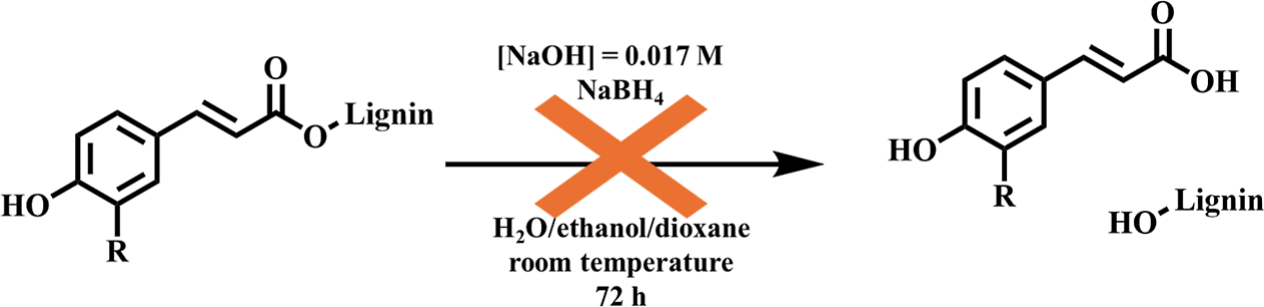
*p*-Coumarate (R = –H) and Ferulate (R = –OCH_3_) Esters Are Not Hydrolysed under PAR Conditions

With regard to the aromatic signals, no relevant variations were observed, leading to an almost unchanged S/G ratio in the sample after the reduction. The ^31^P NMR analyses of the phenolated moieties did not significantly vary, suggesting that the no additional transformations occurred. The GPC analyses of both blank and reduced OL (Figure S8, Supporting Information) demonstrated a slight polymerization, as in other analyzed cases, due to the effect of alkaline solvent. Sodium tetrahydroborate did not affect tricin (T), whose presence was found in both pristine and reduced OL in the very same amount, as both shown by HSQC (Table 5) and ^31^P NMR analyses.

## Conclusions

In the present study, the estimation of carbonyl groups in lignins was explored for the first time using ^31^P NMR analysis, following their selective reduction with sodium tetrahydroborate. A general semimicroscale process (PAR), suitable for a wide variety of lignins (kraft, organosolv, dioxane acidolysis, and enzymatically mild acidolysis), was developed to quantitatively reduce all aldehydic and ketonic carbonyl groups to aliphatic hydroxyl groups. The amount of carbonyl groups was determined by estimating the increase in the content of aliphatic groups via ^31^P NMR. Carbonyl contents estimated with the PAR method were compared to those from the oximation method and quantitative ^13^C NMR, demonstrating consistent results. Unlike oximation, PAR allows for the selective quantification of aldehydic and ketonic carbonyls, as demonstrated by quantitative ^13^C NMR analyses. Finally, the qualitative analyses of the carbonyl groups present in the samples were made via comparison of the HSQC of reduced with pristine lignins, allowing the identification of the nature of different carbonyl groups in the analyzed lignins.

All in one, quantitative ^31^P NMR after sodium tetrahydroborate reduction constitutes a reliable and straightforward analytical protocol for identification and quantification of carbonyl groups in lignin.

## Supplementary Material



## Notes

^31^P NMR spectra of SKL, HKL, and OL before and after reduction, pictorial comparison of the ^13^C NMR spectra of SKL before and after reduction, and HSQC spectra of starting lignin and reduced counterpart as well as the GPC-chromatograms (PDF)
