# Supplementary material for: Quantification of Carbonyl Groups in Lignin: 31P NMR after Reduction
Source: ACS Sustain Chem Eng. 2025 Sep 4;13(36):15189–200. doi: 10.1021/acssuschemeng.5c06762 (PMC12442502; doi:10.1021/acssuschemeng.5c06762)
Supplement: Supplementary file 1 [file sc5c06762_si_001.pdf]

Quantification of Carbonyl Groups in Lignin: ^{31}P NMR after reduction

Nicolò Pajer[a,b] and <u>*Claudia Crestini*</u>*[a,b]

[a] Ca' Foscari University of Venice, Department of Molecular Sciences and Nanosystems, Via Torino 155, Venezia-Mestre, Venice, 30155, Italy.

[b] Centre for Colloid and Surface Science, Via della Lastruccia 3, Sesto Fiorentino, Florence, 50019, Italy.

<u>Pages S1-S9</u>

<u>SUPPORTING INFORMATION</u>

Spectral processing criteria for obtaining comparable results...S2
^{31}P NMR spectra of SKL, HKL, and OL before and after quantitative reduction with $NaBH_4$ S3
^{13}C spectra comparison before and after quantitative reduction S3
HSQC spectra. S4
GPC analyses. S7

<u>LIST of FIGURES INCLUDED</u>

^{31}P NMR spectra of SKL, HKL, and OL before and after quantitative reduction with sodium tetrahydro borate;
^{13}C NMR spectra of SKL before and after quantitative reduction with zooms in the carbonyl regions;
Figures S1-S3 HSQC spectra of SKL, HKL, and OL before and after quantitative reduction;
Figures S4-S6 chromatograms of SKL, HKL, and OL, blanks, and reduced counterparts using different sodium tetrahydroborate loadings.

Spectral processing criteria for obtaining comparable results.

The following considerations were taken in consideration while performing the spectral processing and calculations resulting in the estimation of the content of aliphatic hydroxyl groups ensuring good reproducibility of the results:

1. the phasing has to be carefully performed after applying the *Fourier* transformation to the spectra; the use of sub-routinary phase programmes available in the software controlling the NMR spectrometer is recommended;[1]
2. a minimum baseline correction is suggested due to the high variability of the integrals;[1,2]
3. apodization operations, allowing to increase the signal-to-noise ratio, are not recommended. They drastically affect the integrals resulting in unreliable results. The amount of sample utilised for the analyses (~20 mg) proved to be enough to acquire a good and processable quality spectrum using a 300 MHz instrument. In the case of 200 MHz spectrometer, as well as in the case of bench-top instruments, ~30 mg samples demonstrated to be suitable for obtaining processable and meaningful spectra.[3]

References:

(1) Robert, D. Carbon-13 Nuclear Magnetic Resonance Spectrometry. In *Methods in Lignin Chemistry*; Lin, S. Y., Dence, C. W., Eds.; Springer Series in Wood Science; Springer: Berlin, Heidelberg, 1992; pp 250–273. https://doi.org/10.1007/978-3-642-74065-7_18.

(2) Pajer, N.; Danelon, U.; Crestini, C. NMR Spectroscopy: An Invaluable Tool to Identify Lignin Structural Modifications Induced by Oxidative Enzymes. In *Lignin-Degrading Enzymes*; Methods in Enzymology; 2025; Vol. 716.

(3) Araneda, J. F.; Burton, I. W.; Paleologou, M.; Riegel, S. D.; Leclerc, M. C. Analysis of Lignins Using 31P Benchtop NMR Spectroscopy: Quantitative Assessment of Substructures and Comparison to High-Field NMR. *Can. J. Chem.* **2022**, *100* (11), 799–808. https://doi.org/10.1139/cjc-2022-0041.

^{31}P NMR spectra of SKL, HKL, and OL before and after quantitative reduction with $NaBH_4$

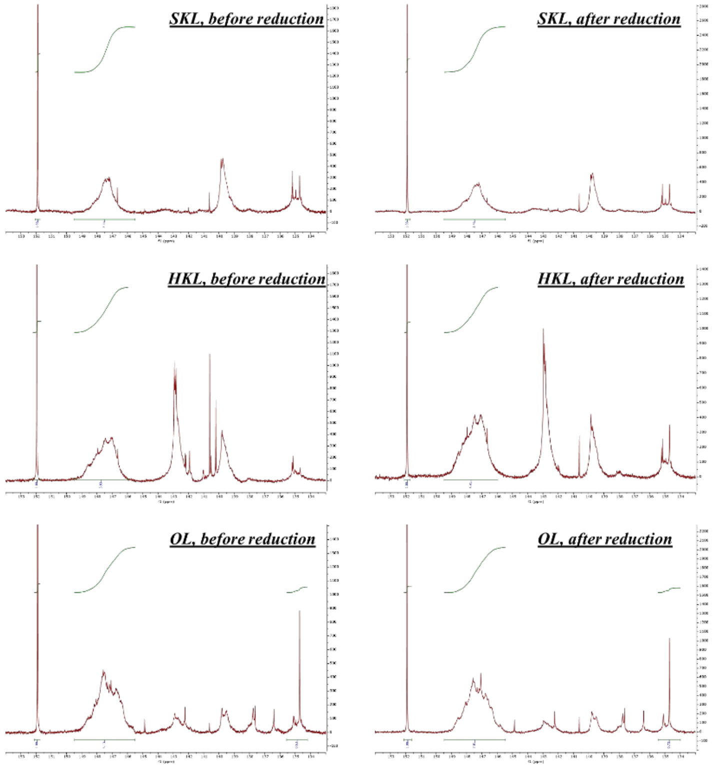

^{13}C spectra comparison before and after quantitative reduction

SKL, before quantitative reduction

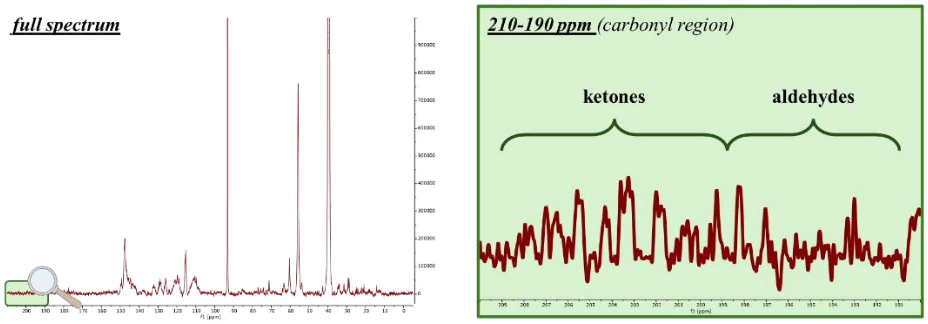

SKL, after quantitative reduction

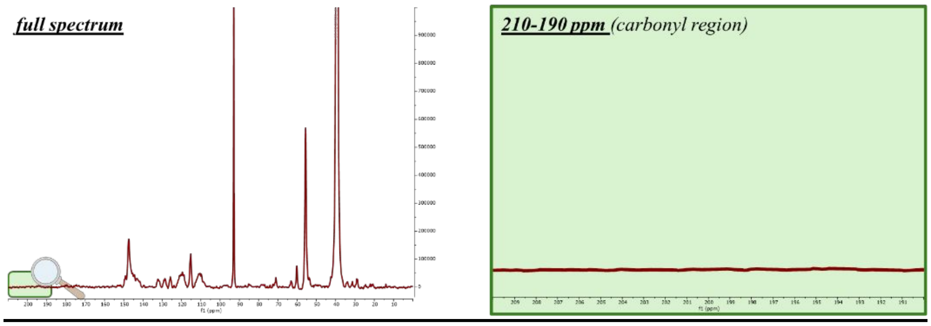

HSQC spectra.

Bonding patterns referred in the HSQC spectra with letters correspond to the following structures:

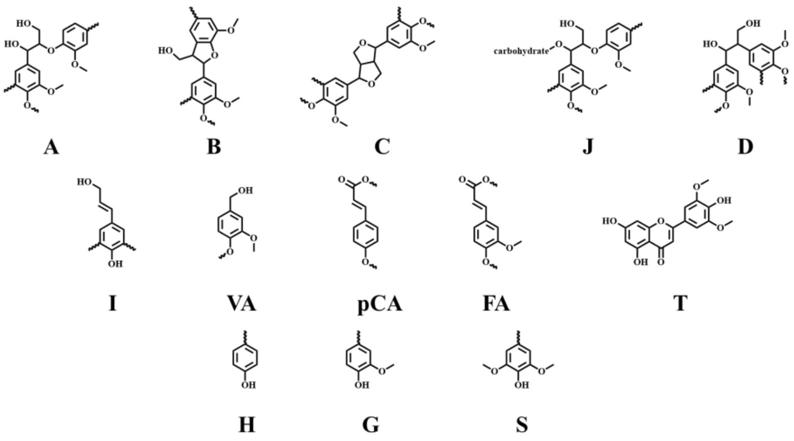

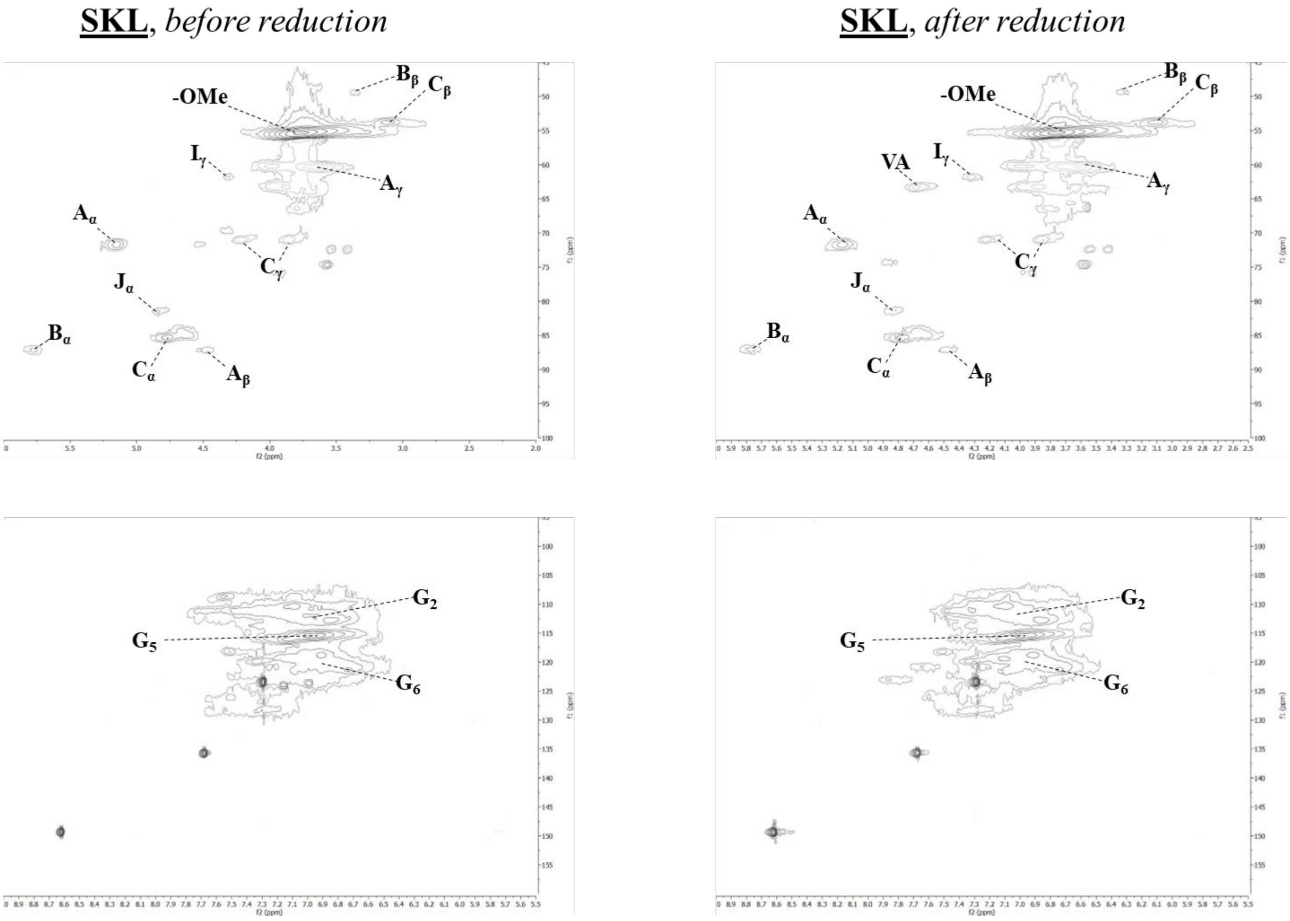

Figure S1. HSQC spectra of pristine and sodium tetrahydroborate reduced SKL. (*up*, oxygenated area; *down*, aromatic area).

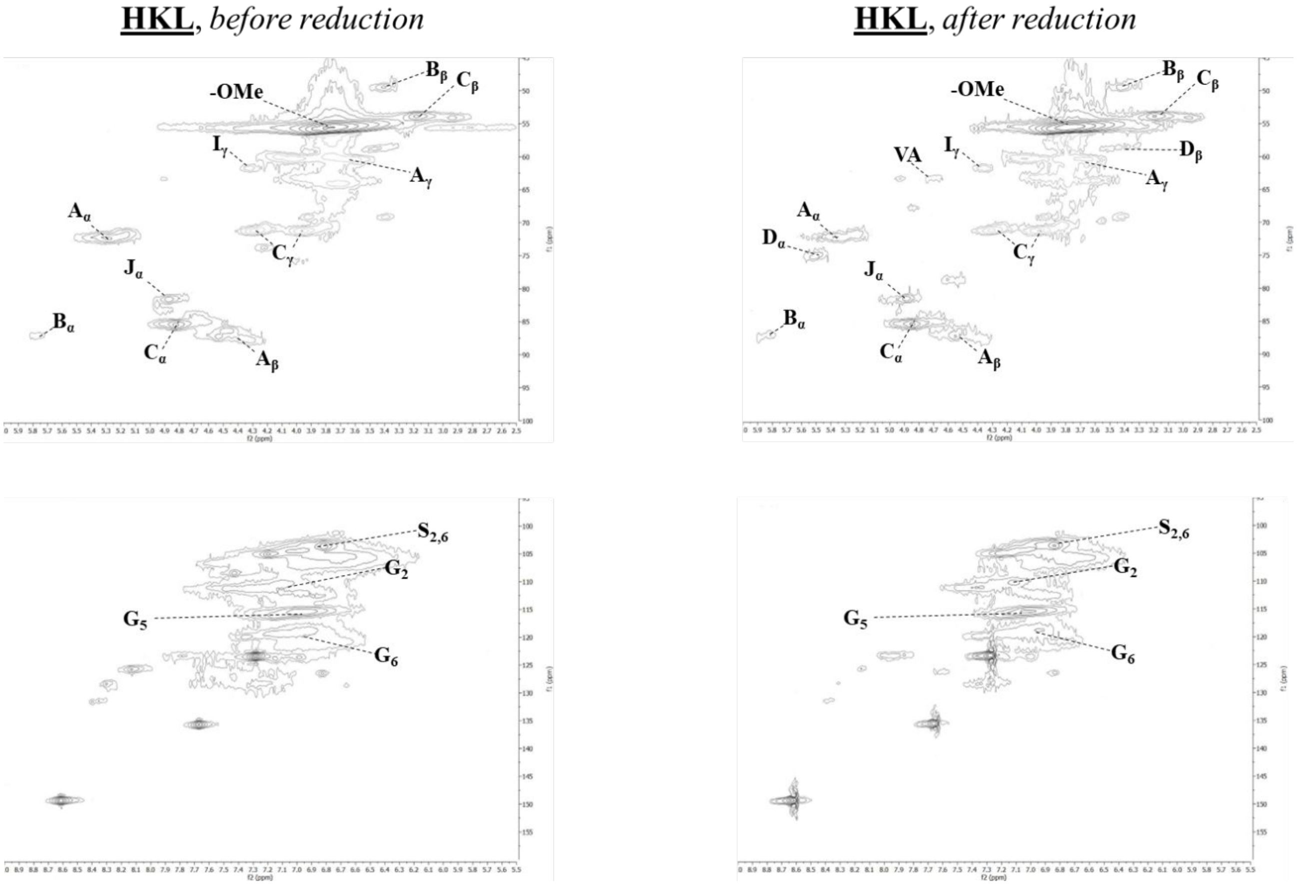

Figure S2. HSQC spectra of pristine and sodium tetrahydroborate reduced HKL. (*up*, oxygenated area; *down*, aromatic area).

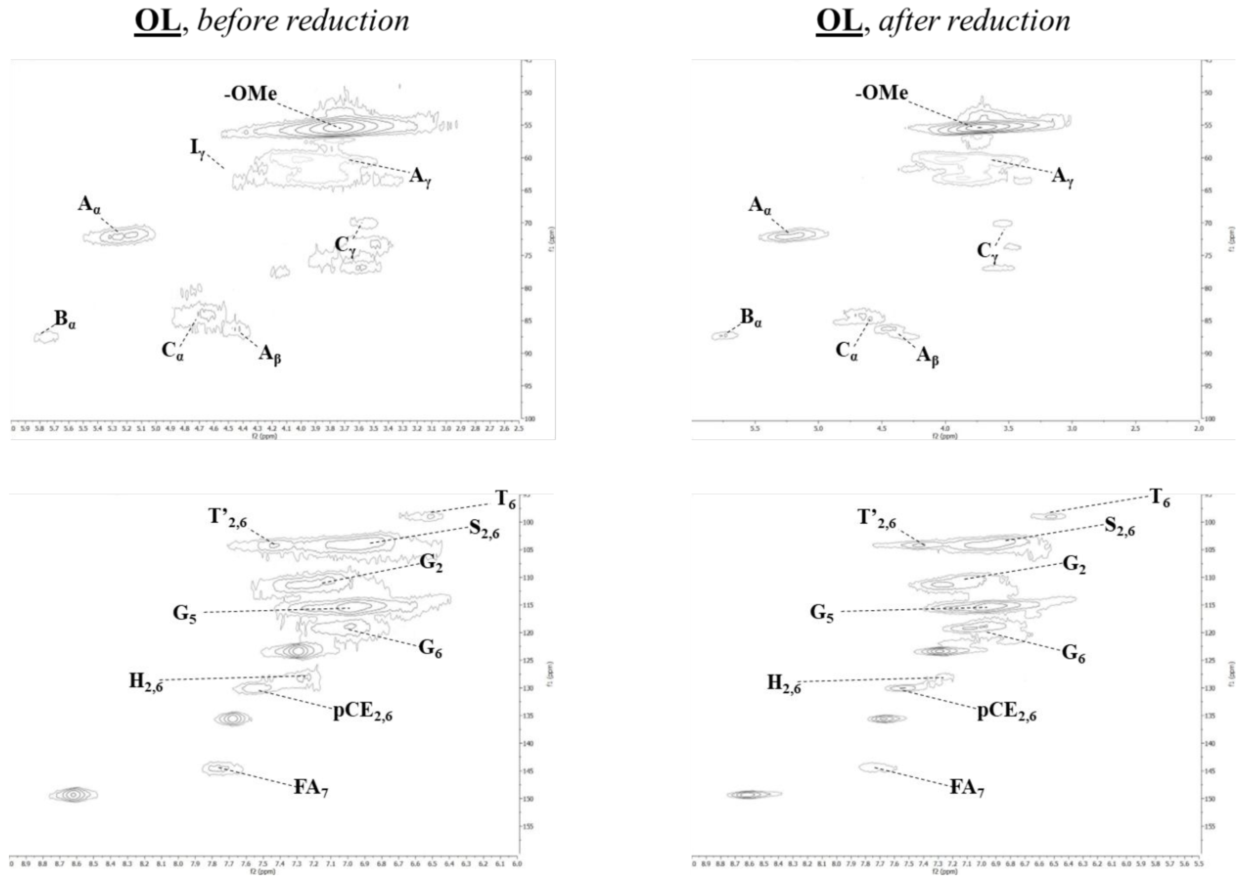

Figure S3. HSQC spectra of pristine and sodium tetrahydroborate reduced HKL. (*up*, oxygenated area; *down*, aromatic area).

GPC analyses.

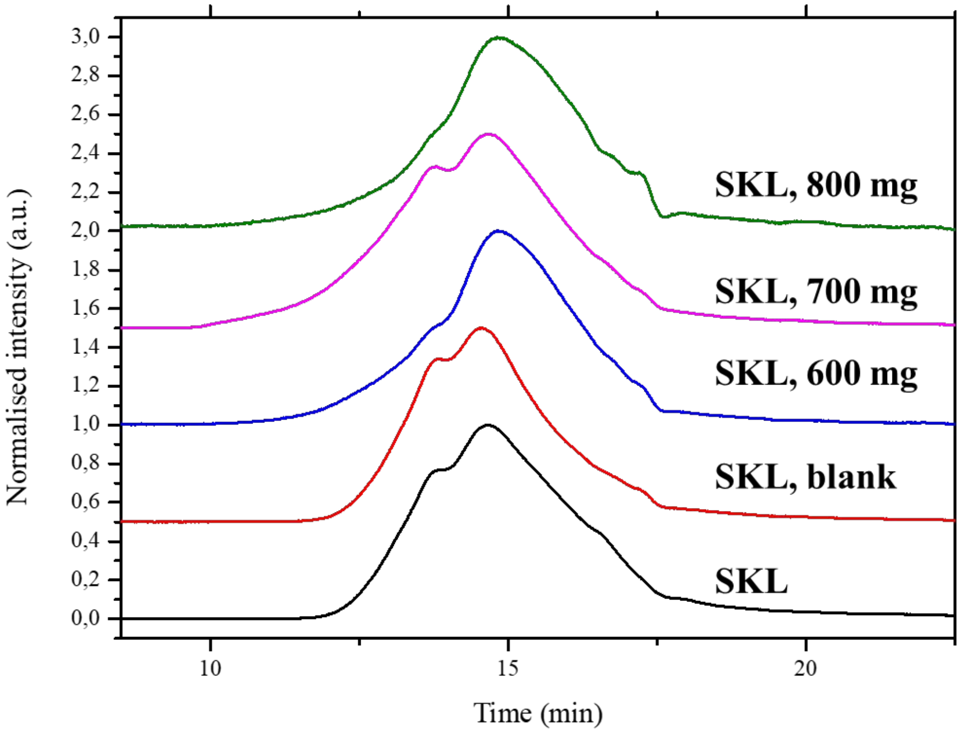

Figure S4. GPC eluograms for pristine SKL, SKL blank in NED, SKL reduced with different amounts of sodium tetrahydroborate.

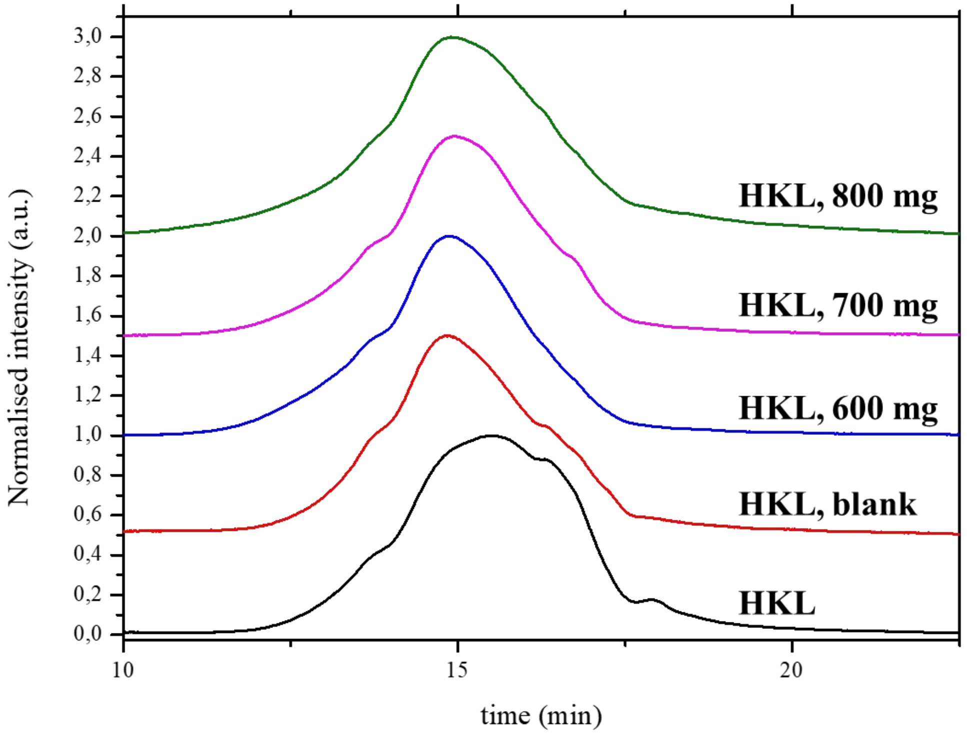

Figure S5. GPC eluograms for pristine HKL, HKL blank in NED, HKL reduced with different amounts of sodium tetrahydroborate.

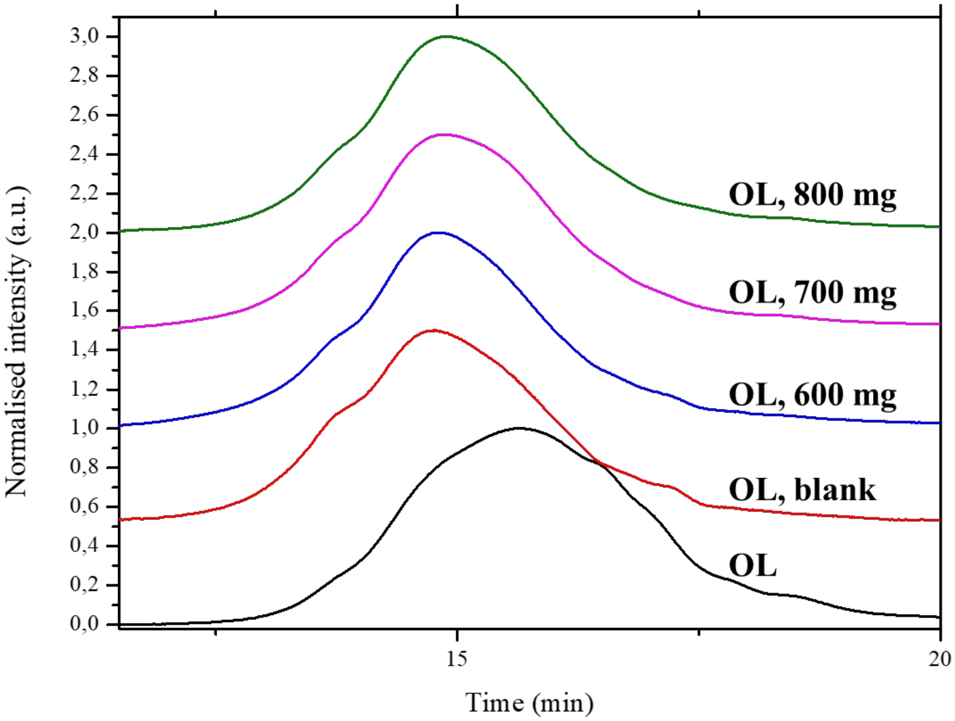

Figure S6. GPC eluograms for pristine OL, OL blank in NED, OL reduced with different amounts of sodium tetrahydroborate.
